# Label‐Free Multiphoton Endomicroscopy for Minimally Invasive In Vivo Imaging

**DOI:** 10.1002/advs.201801735

**Published:** 2019-02-25

**Authors:** Ashwathama Dilipkumar, Alaa Al‐Shemmary, Lucas Kreiß, Kristian Cvecek, Birgitta Carlé, Ferdinand Knieling, Jean Gonzales Menezes, Oana‐Maria Thoma, Michael Schmidt, Markus F. Neurath, Maximilian Waldner, Oliver Friedrich, Sebastian Schürmann

**Affiliations:** ^1^ Institute of Medical Biotechnology Friedrich‐Alexander‐Universität Erlangen‐Nürnberg (FAU) Paul‐Gordan‐Str. 3 91052 Erlangen Germany; ^2^ Erlangen Graduate School in Advanced Optical Technologies Friedrich‐Alexander‐Universität Erlangen‐Nürnberg (FAU) Paul‐Gordan‐Str. 7 91052 Erlangen Germany; ^3^ Institute of Photonic Technologies Friedrich‐Alexander‐Universität Erlangen‐Nürnberg (FAU) Konrad‐Zuse‐Str. 3–5 91052 Erlangen Germany; ^4^ Department of Internal Medicine 1 University Hospital Erlangen Ulmenweg 18 91054 Erlangen Germany; ^5^ Department of Pediatrics and Adolescent Medicine University Hospital Erlangen Loschgestr. 15 91054 Erlangen Germany

**Keywords:** endoscopy, inflammatory bowel disease, multiphoton microscopy, optical imaging

## Abstract

Multiphoton microscopy of cellular autofluorescence and second harmonic generation from collagen facilitates imaging of living cells and tissues without the need for additional fluorescent labels. Here, a compact multiphoton endomicroscope for label‐free in vivo imaging in small animals via side‐viewing needle objectives is presented. Minimal invasive imaging at cellular resolution is performed in colonoscopy of mice without surgical measures and without fluorescent dyes as a contrast agent. The colon mucosa is imaged repeatedly in the same animal in a mouse model of acute intestinal inflammation to study the process of inflammation at the tissue level within a time period of ten days, demonstrating the capabilities of label‐free endomicroscopy for longitudinal studies for the first time.

## 1 Introduction

Microscopic examination of organs and tissues is essential for diagnostics and research in many pathologies including cancer and chronic inflammatory or degenerative diseases. In clinical practice, the standard diagnostic procedure for microscopic assessment is still the acquisition of biopsies followed by mechanical sectioning and histological staining methods. This might include frequent biopsies in the case of chronic diseases, with an associated risk of wound‐healing problems, and a delay of diagnostics, in general. There is a large interest to evaluate cellular processes in living animals, for example, to study the development of tumors, the process of inflammation, or assessment of genetically modified small animals.

For these reasons, there has been a long‐standing interest in exploring endomicroscopy techniques applicable to preclinical and clinical settings. The final aim is to acquire histological information in vivo without the need for biopsies or surgical interventions.

Confocal laser‐scanning endomicroscopy has demonstrated the ability to image tissue in vivo with cellular resolution. It found application in a number of preclinical studies, for example, to visualize blood vessels in murine colon,^1^, ^2^ and also in clinical studies, for example, during colorectal cancer screening endoscopy,^3^ or as a molecular imaging approach using fluorescence‐labeled antibodies to predict therapeutic responses in inflammatory bowel disease patients.^4^ However, several limitations confine its practical usage, such as the general requirement for fluorescent dyes as a contrast agent, as well as the limited imaging depth due to light scattering in tissue.

Multiphoton microscopy (MPM) offers several advantages in this regard:^5^ i) The use of near‐infrared laser pulses, which are less prone to scattering and enhance the imaging depth. ii) The nonlinear excitation process is confined to the small focal volume of highest energy density, which allows for thin optical sectioning and confocal resolution without the use of a pinhole. iii) Tissue imaging is possible without the application of external fluorescent dyes (label‐free), entirely based on autofluorescence (AF) from endogenous molecules, such as nicotinamide adenine dinucleotide (NADH) or flavin adenine dinucleotide (FAD), and second harmonic generation (SHG) from collagen‐I, myosin‐II, or tubulin. Commercial and custom‐built MPM setups are available in many research institutions today. In the field of dermatology, MPM is also available for label‐free tissue imaging of human skin in vivo in clinical applications.^6^

MPM in endoscopic settings has been demonstrated in proof‐of‐concept studies, implementing two basic approaches: a) scanning microscopes with standard‐sized optical components and objectives coupling into a rigid needle endoscope made from gradient index (GRIN) lenses,^7^, ^8^ and b) fully miniaturized resonant fiber‐scanning endomicroscopes.^9^, ^10^, ^11^ Here, we further explore the potential of the technology for minimal invasive in vivo imaging. We have developed an advanced multiphoton endomicroscope, combining side‐view endoscope optics, with multiphoton excitation and label‐free detection, further adding 3D sectioning capabilities to the system by means of a fast electrically tunable lens (ETL). Compared to previous studies, we have applied multiphoton endomicroscopy in a truly minimal invasive manner without any surgical measures, and imaged the colon mucosa repeatedly in the same animal during intestinal inflammation, demonstrating the capabilities of label‐free endomicroscopy for longitudinal studies for the first time.

## 2 Results

### 2.1 Multiphoton Endomicroscope

**Figure 1:**
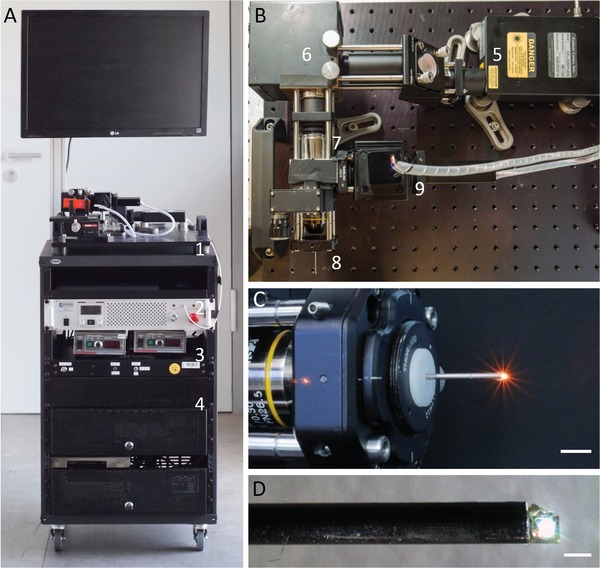
Multiphoton endomicroscope in a compact custom‐built setup. A) Transportable standalone system including (1) scanning microscope, (2) laser, (3) electronics, and (4) PC. B) Top view of the endomicroscope showing (5) laser head, (6) laser scanner, scan lens, and (7) tube lens, (8) coupling and endomicroscope objective, and (9) PMT detectors. C) Coupling objective and mounted endomicroscope objective. Scale bar: 1 cm. D) Endomicroscope objective with front‐mounted prism for side view. Scale bar: 1 mm. For in vivo experiments, the objective was mounted inside a 1.4 mm steel cannula and a droplet of glue was added to the back of the prism to cover its sharp edges and facilitate smooth positioning during colonoscopy.

Our compact standalone system is entirely rack‐mounted and easily transportable between laboratories and animal facilities (**Figure** **1**). It includes a 780 nm femtosecond fiber laser, galvanometric scanning mirrors, and an ETL for fast shifting (response time < 20 ms) of the focal plane and acquisition of 3D volume stacks. A detailed technical description of the engineering of our multiphoton endomicroscope is provided in Experimental Section.

**Figure 2:**
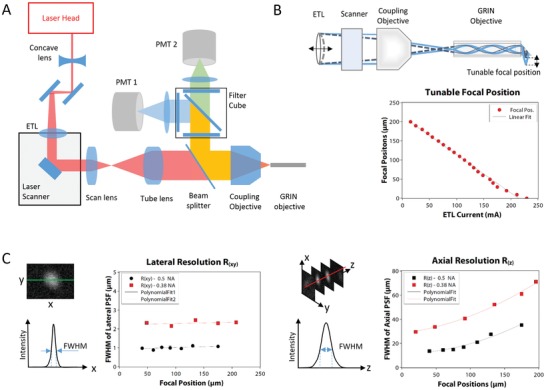
Optical setup and performance. A) Schematic drawing of the optical path from laser head to endoscope objective and back to PMT detectors. See Experimental Section for further details. B) Illustration of focal plane shifting by means of an ETL and dependency of the focal position on the actuation current. C) Lateral and axial resolution measured as the size of the point spread function of 30 nm sized fluorescent beads dependent on the focal position.

We have manufactured a number of different needle objectives for endomicroscopy based on GRIN lenses. The images presented in this study were recorded with an endoscope objective of 32 mm length and 1.4 mm diameter (including a stainless steel housing) that features a reflective prism for side view at the front tip (Figure 1C), similar to previously reported designs for confocal endomicroscopes.^1^, ^12^ For in vivo experiments, a droplet of glue was added to the back of the prism to cover its sharp edges and facilitate smooth positioning during colonoscopy. Inside the mouse, the surface of the prism remained in direct contact with the colon wall. The mucosal tissue could be imaged in three dimensions by raster‐scanning the laser beam in the *x–y* plane with a field of view of 290 × 290 µm and shifting the focal spot (**Figure** **2**B) for depth information in a range of 200 µm along the *z*‐axis using the ETL. The achievable resolution for the side‐view endoscope, which had a calculated numerical aperture of *NA* = 0.38, was 2.2–2.5 µm (lateral) and 30–60 µm (axial). This resolution in *z*‐direction is due to effects of the small dimensions, the front prism, and by well‐known limitations of the ETL. In tissue imaging applications, however, these effects were not disruptive.

### 2.2 Endomicroscopic In Vivo Imaging

**Figure 3:**
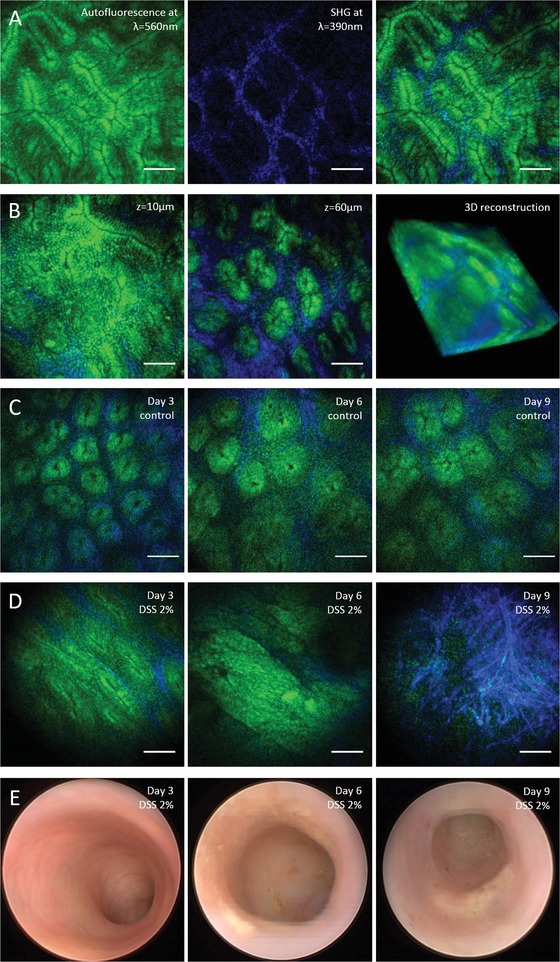
Label‐free multiphoton endomicroscopy of murine colon mucosa in vivo. A) Two‐photon excited AF from endogenous molecules such as NADH or FAD (shown in green) and SHG from collagen‐I (shown in blue) show the epithelial crypt structure and underlying lamina of the colon mucosa. B) Focal plane shifting by means of a fast ETL for 3D visualization of the tissue morphology. C) Repeated minimal invasive multiphoton endomicroscopy of healthy murine colon as a control for the colitis model. D) Changes in the tissue morphology during the time course of intestinal inflammation in a mouse model of ulcerative colitis ranging from deformations of the crypt pattern at day 3, to massive epithelial erosion and collagen matrix formation at day 9. Images were recorded in the same animal with minimal invasive multiphoton endomicroscopy. E) Conventional wide‐field endoscopy as reference showing clear symptoms of acute murine DSS colitis from day 6 to 9, and macroscopically unchanged mucosa at day 3. Scale bars: 50 µm.

Label‐free tissue imaging was performed by recording two color channels, one for AF and one for SHG signals from collagen. In colonoscopy of mice (**Figure** **3**), we were able to clearly resolve the crypt pattern in vivo in both color channels, as shown in Figure 3A, by AF from epithelial cells inside the crypts (green) and SHG signals surrounding the crypts (blue). The focal plane could be quickly shifted from inside the epithelial layer to inside the underlying lamina propria (Figure 3B). Volume stacks could be recorded for 3D reconstruction of the tissue. Considering the facts that no fluorophores were used and imaging was performed in vivo through a thin needle objective, the system provides high image quality and allows a detailed visualization of the crypt morphology.

We applied label‐free multiphoton endomicroscopy to study the time course of inflammation in the murine colon in a model of acute colitis using 2% dextran sodium sulfate (DSS) in the drinking water for 9 days. Repetitive minimal invasive imaging of the same mice was performed on days 3, 6, and 9. Label‐free multiphoton imaging (Figure 3C,D) was applied along with conventional white light wide‐field endoscopy (Figure 3E), as a well‐established technology for macroscopic imaging of the murine colon mucosa,^13^, ^14^, ^15^ in order to study both macroscopic and microscopic changes in tissue morphology.

Conventional endoscopy showed typical signs of inflammation, such as increased granularity, fibrin deposits, decreased translucency, and pathologic vascularity, as described in previous studies,^13^, ^14^ indicating that inflammation of the colon progressed as expected in this mouse model of colitis (Figure 3E). With label‐free multiphoton endomicroscopy, we were able to study inflammation of the colon mucosa at the tissue level with cellular resolution. Before DSS treatment and in healthy control mice (Figure 3A,B), also after repeated colonoscopy (Figure 3C), the epithelial layer was intact, and crypts were of circular shape and similar sizes arranged in a pattern with regular crypt distances (see Figure 3C). In the progress of DSS‐induced inflammation, we observed numerous changes in tissue morphology (Figure 3D). At day 3, the distance between the crypts appeared larger and the distribution less isotropic, the shape of the crypts was more elliptical. At day 6, crypts appeared largely deformed, the regular crypt pattern was lost, and in some regions, no more crypts were visible in the field of view. Furthermore, an erosion of the epithelial layer was apparent in several images, and the collagen matrix showed an altered structure compared to before administration of DSS. At day 9, a pronounced increase in SHG signals from collagen was observed, in parallel with a loss of distinct structures in the AF channel, indicating that the regular mucosal epithelium has considerably degenerated and been partly replaced by scar tissue with high content of extracellular matrix. Overall, it was possible to study the time course of acute inflammation of the colon mucosa at the microscopic scale in vivo in the same animal.

**Figure 4:**
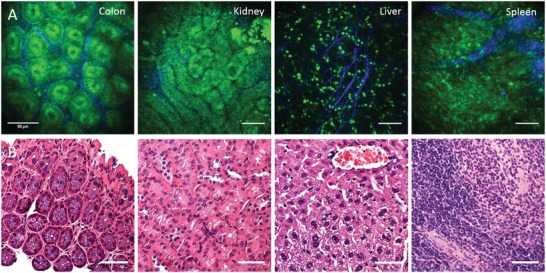
Label‐free endoscopic imaging of different organs ex vivo. A) Murine organs imaged ex vivo through a side‐view endomicroscope objective. B) Histological sections stained with haematoxylin and eosin (H&E) for comparison. Scale bars: 50 µm.

To confirm the general applicability of label‐free endomicroscopy, we also analyzed fresh tissue from other organs, such as kidney, liver, and spleen ex vivo (**Figure** **4**). Images from the kidney clearly show the renal tubules by AF, and also surrounding collagen matrix. In the liver, the fine network of the outer collagen matrix of the fibrous capsule is apparent in the SHG channel, while several distinct bright spots appear in the AF channel. It is believed that these spots are associated with Kupffer cells.^16^ Imaging of spleen showed AF from individual cells and a fibrous structure in the SHG channel, representing the typical trabeculae of the spleen. The label‐free images of kidney, liver, and spleen tissue show that multiphoton endomicroscopy is also suitable to study these organs in vivo. Although in contrast to colonoscopy, small surgical incisions are necessary here, multiphoton endomicroscopy experiments may still be carried out in a minimal invasive way, compared to state‐of‐the‐art two‐photon in vivo imaging with current research microscopes.

## 3 Discussion

Multiphoton endomicroscopy allows to study organs in vivo at the tissue level. With label‐free detection based on AF from endogenous molecules, such as NADH and FAD, and SHG from collagen, native tissue is visualized without previous treatment or processing, which opens an interesting perspective for future application in clinical diagnostics and therapy monitoring. Label‐free imaging will therefore be of increasing relevance also in studies of mouse models of human disease, which offer many additional detection modalities for multiphoton endomicroscopy by means of highly specific fluorescent markers or fluorescent protein expression that are generally not available for in vivo imaging in humans. As shown in Figures 3 and 4, label‐free multiphoton imaging provides a detailed visualization of the tissue morphology in different organs and can further identify inflammation‐related morphological changes at the tissue level with cellular resolution.

The multiphoton endomicroscope that we engineered for this study allows to record these images in vivo in mice via thin needle objectives. Compared to previous studies,^7^ we have now established a setup that works in a truly minimally invasive way in a colonoscopy setting without surgical measures. It takes recordings with minimal effort, as the side‐viewing front optic of the endoscope is in direct contact with the colon wall during experiments and information from different tissue layers is quickly accessible by means of an ETL. The image quality in vivo is sufficient to examine tissue with cellular resolution based on AF and SHG. Due to the small dimensions of the endoscope objective (1.4 mm diameter over a length of 32 mm) and the limited numerical aperture (*NA* = 0.38) in side‐view geometry, however, it is not directly comparable to stained histological sections or to label‐free multiphoton imaging using research microscopes with state‐of‐the‐art water immersion objectives (*NA* = 1.0), as demonstrated in previous ex vivo studies.^17^, ^18^, ^19^ Image acquisition was generally fast and straightforward, the colon mucosa stayed in focus, and areas of interest remained in the field of view with our side‐viewing endoscope objective. The ETL allowed fast focus shifting from epithelium to lamina propria between alternating frames, which effectively generated a two‐layer live scanning mode at half frame rate that proved particularly useful for in vivo imaging applications. The maximum imaging depth in colon tissue was roughly 120 µm. The imaging frame rate was in the range between 2 and 0.5 fps, with activated line averaging and dual layer scan. Motion artifacts caused by intermittent bowel movement were noticable in the course of all in vivo experiments, for example, as shifts in the field of view between frames, or distortions along the slow scanner axis in individual images. Our impression is, however, that the overall usability of the device is not substantially impaired by these issues. As a future direction, we intend to address the problem of motion artifacts with compensation algorithms, as demonstrated in other intravital microscopy studies.^20^, ^21^, ^22^

We applied label‐free multiphoton endomicroscopy in vivo to investigate the time course of acute inflammation of the colon mucosa at the microscopic scale in the same animal. In a mouse model of acute colitis, we visualized deformation of the regular crypt structure, erosion of the epithelium, and collagen matrix formation without fluorescent markers. Changes in the colon mucosa were apparent earlier in the time course of inflammation, compared to conventional endoscopy. The next step of technical development will be the integration of conventional white light wide‐field endoscopy and multiphoton endomicroscopy, and in a single device combining the modalities for a large field of view with those for label‐free tissue imaging at cellular resolution. Additional detection channels for fluorescent proteins will be included into the setup to study pathways of inflammation in transgenic mouse models. Other modalities for label‐free optical detection, such as coherent anti‐Stokes Raman scattering (CARS) microscopy, optical coherence tomography (OCT), optoacoustic imaging, or molecular spectroscopy methods including infrared and Raman spectroscopy,^23^, ^24^, ^25^, ^26^, ^27^, ^28^, ^29^ could further complement the technology and direct it toward a multimodal endoscopy approach in the future.

## 4 Conclusion

Overall, we have engineered a multiphoton endomicroscope for label‐free examination of tissue in a truly minimally invasive setting comparable to regular colonoscopy. We expect broad applicability of multiphoton endomicroscopy for in vivo tissue imaging in a range of research studies, and further see the potential to develop label‐free imaging for clinical endomicro‐scopy in the long‐term perspective.

## 5 Experimental Section

*Multiphoton Endomicroscope*: The setup was entirely mounted on a 19″ transport rack, including laser, electronics, PC, monitor, and optics, thus making the endomicroscope portable to clinics and laboratories for in vivo studies. The optical components of the endomicroscope were set up on the top plate of the rack on an optical breadboard (45 × 45 cm), as shown schematically in Figure 2. The laser was a compact, high‐power, mode‐locked fiber laser (Carmel, Calmar Laser, Palo Alto, USA), covering two high units on the rack. The laser head (18 × 9 cm) was placed on the breadboard and delivered a collimated free‐space propagating beam of up to 500 mW at 780 nm. The laser emitted pulses of 80 fs pulse duration (full width at half maximum (FWHM)) at a repetition rate of 50 MHz. A low reflectivity shutter (SHB1T, Thorlabs, Newton, USA) was installed at the exit aperture of the laser and controlled remotely via software.

For fast shifting of the focal plane, an ETL (Optotune AG, Dietikon, Switzerland) was integrated into the setup. The focal length of the ETL was tunable between *f* = 202 mm and *f* = 36 mm, depending on the actuating current. In combination with a concave lens (*f* = −50 mm), the ETL formed a threefold beam telescope. Behind the ETL, the laser beam was centered onto a pair of galvanometric mirrors (6210H, Cambridge Technology, Bedford, MA), which were used for lateral scanning. Two telecentric lenses with short effective focal lengths represented scan lens (LSM02‐BB, EFL = 18 mm, Thorlabs) and tube lens (LSM03‐BB, EFL = 36 mm, Thorlabs) in a 4f‐configuration expanding the beam twofold. A 10× objective (Plan Fluorite RMS10X‐PF, Olympus, Tokyo, Japan) with numerical aperture of *N.A.* = 0.3 was used to focus and point‐scan the laser in the back focal plane of the endoscope objective.

Several endoscope objectives were manufactured from GRIN lenses. In particular, two designs had been calibrated that are displayed in this article: a standard GRIN objective with straight view (NEM‐100‐25‐10‐860‐DL‐ST, Grintech, Jena, Germany), and an in‐house designed GRIN objective with mirror prism for side view. This side‐view GRIN objective was a combination of two laser focusing rod lenses (GT‐LFRL‐100‐125‐20‐NC and GT‐LFRL‐100‐013‐50‐NC, Grintech, Jena, Germany) as relay and objective lenses, respectively. Side‐view imaging was realized by a 1 mm sized prism, which was glued to the front of the objective lens. In either case, the GRIN objective served as main component of the rigid endoscope head that was inserted into the animal. The distance between GRIN and coupling objective was set up such that the focal range started at the prism surface and extended into the tissue (Figure 2B).

AF and SHG signals from the tissue were collected by the endoscope objective and guided back through the 10× objective. A dichroic beam splitter (HC 705 LP, Semrock, Rochester, NY, USA) was installed between the beam expander and the objective in order to filter all wavelengths above 705 nm. Light with shorter wavelength was reflected to the detection arm, mainly consisting of a filter cube with appropriate filters and photomultiplier tube (PMT) modules. The foremost filter (ET680SP‐2P8, Chroma, Bellow Falls, VT, USA) was used to block additional residues of the excitation wavelength at 780 nm. Subsequently, a beam splitter was used to split the signal to two imaging channels. However, the system can be further extended to a three channel imaging system by the use of a second beam splitter, a third photomultiplier tube (PMT), and the corresponding combination of filters. The results presented here were recorded with a dichroic beam splitter at 495 nm (HC BS 495, Semrock), and the two filters (525/50 HC and 387/11 BrightLine HC, Semrock) were chosen in order to target AF of NADH and FAD in the range of 500–550 nm and the SHG signal at 390 nm. Finally, a photomultiplier tube (H10770‐40, Hamamatsu, Japan) with associated preamplifiers detected the signals in each channel.

*Data Acquisition and Software Control*: The endomicroscope was controlled by a scan software with a graphical user interface (GUI) developed and implemented in‐house using MATLAB. The software controlled the shutter, the ETL, as well as the galvanometric scanner, and enabled several different scan modalities such as uni‐ or bidirectional scanning, frame or line averaging, and a two‐layer scan. Furthermore, it received the data from the PMT modules, reconstructed the images, and saved them as bioformat files (OME.TIF), supplemented by all relevant metadata. The communication between the GUI and the endomicroscope was established by a multifunction data acquistion module (PXIe‐6366, National Instruments, Austin, TX, USA). The card provided two analog outputs that were used to control the galvanometric scan mirrors. Simultaneously, two analog inputs were used for the detected signal from the photomultipliers. The ETL was controlled by a programmable DC power supply (PXI‐4110, National Instruments) that communicated with the MATLAB software. The presented 3D reconstructions had been rendered by the “3D Viewer” plugin in ImageJ after image registration and size adjustment to obtain cubic voxels.

*Optical Characterization*: The optical performance of the endomicroscope and effects of the ETL were characterized by measuring the resolution using 30 nm sized fluorescent beads (L5155, Sigma‐Aldrich, St. Louis, MO), embedded in 1% agarose. All measurements were based on the FWHM of the intensity profiles (Figure 2C). The axial focal position of the system with respect to the ETL was calibrated using sub‐resolution beads imaged at multiple *z*‐positions in steps of 5 µm, by using a mechanical stage. At every step, a full range *z*‐scan was performed via the ETL. An intensity profile across the acquired *z*‐stack allowed to find the location of the focal plane. This location was correlated to the electrical current controlling the ETL, as seen in Figure 2B. The results were used as a look‐up table in the software to calibrate ETL current values to distance values with respect to the last surface of the GRIN objective. The axial resolution was characterized with the same procedure; a full *z*‐scan of subresolution beads was performed each time after mechanically moving the beads by 5 µm. The intensity profile was extracted across the axial direction and the FWHM of the Gaussian profile was estimated to be the axial resolution *R_z_*. The lateral resolution at the corresponding focal positions of the ETL was deduced from an *x–y* scan of the focal plane wherein several beads were imaged. An intensity profile across each bead was extracted and the respective FWHM was deduced. The averaged FWHM across all beads in the frame was taken to be *R_xy_*. The effect of the ETL on the resolution is displayed in Figure 2C. From the results, it was clear that the ETL had a significant effect on the axial resolution with 15–30 µm for the front‐view GRIN and 30–60 µm for the side‐view objective. Compared to that, the effect on the lateral resolution is only minor, varying between 0.9 and 1.2 µm for front view and 2.2–2.5 µm for side view. This behavior represents the performance of ETL configurations and can be explained by aberrations induced by the ETL,^30^ which prevent diffraction‐limited performance.

*In Vivo Mouse Colonoscopy and Colitis Model*: All animal experiments were conducted at the Department of Internal Medicine 1, University Hospital Erlangen, in compliance with all institutional guidelines. Throughout the entire session, the animals were anesthetized using a gas mixture of 5 L min^−1^ O_2_ containing 2–4% of isoflurane administered by an anesthetic unit (Isoflurane Vaporizer TEC 3 and Oxyvet oxygenation unit, Eickemeyer, Tuttlingen, Germany). The colon was flushed several times with water prior to the colonoscopy in order to remove remaining excrement residuals. Conventional white light wide‐field endoscopy was performed on a commercial rigid endoscopy system (COLOVIEW Mainz, KARL STORZ Endoscopes, Tuttlingen, Germany). Label‐free multiphoton imaging was carried out on the custom endomicroscope engineered for the present study. Fluorescent markers were not used. Acute colitis was induced in C57Bl/6 mice by providing 2% DSS (MP Biomedicals LCC, Canada) in the drinking water, as previously described.^31^

*Imaging of Mouse Organs*: To confirm the general applicability of label‐free endomicroscopy, fresh tissue was also analyzed from other organs, such as kidney, liver, and spleen ex vivo (Figure 4). The organs were surgically removed and were minimally cut at about 3 mm length, allowing access to the outside and the inside of the tissue. The organs were investigated by placing them on a sample holder below the GRIN objective with the prism surface facing downward and being in direct contact with the sample. The time delay between measurement and surgical removal of the samples was less than 10 min and the organs had not been cooled, fixed, labeled, or treated in any way.

## Conflict of Interest

The authors declare no conflict of interest.
